# Surgical management of anomalous aortic right coronary artery discovered during acute type A aortic dissection: a case report

**DOI:** 10.1093/jscr/rjae348

**Published:** 2024-07-13

**Authors:** Zamaan Hooda, Yasmine Rifai, Elissa LeBow, John Paul Bustamante, Luis Cerda, Bledi Zaku

**Affiliations:** Department of Cardiothoracic Surgery, St. Joseph’s University Medical Center, Paterson, NJ 07503, United States; Department of Surgery, Hackensack Meridian School of Medicine, Nutley, NJ 07110, United States; Department of Cardiothoracic Surgery, St. Joseph’s University Medical Center, Paterson, NJ 07503, United States; Department of Cardiothoracic Surgery, St. Joseph’s University Medical Center, Paterson, NJ 07503, United States; Department of Cardiothoracic Surgery, St. Joseph’s University Medical Center, Paterson, NJ 07503, United States; Department of Cardiothoracic Surgery, St. Joseph’s University Medical Center, Paterson, NJ 07503, United States

**Keywords:** anomalous right coronary artery, type A aortic dissection, coronary artery bypass graft, cardiothoracic surgery

## Abstract

Anomalous aortic origin of the right coronary artery (RCA) is a rare anatomic anomaly that is present in ~1% of the general population, and is often discovered incidentally through imaging performed for another purpose. Despite being an uncommon phenomenon, aberrant right coronary arterial origins can have devastating manifestations in half of affected patients. These include myocardial infarction, arrhythmias, heart failure, syncope, and sudden cardiac death secondary to ischemia of the cardiac tissue. This report describes a case of a 48-year-old female patient that was initially found to have ST-elevation myocardial infarction. During cardiac catheterization, the patient was discovered to have a type A aortic dissection. Cardiothoracic surgery was consulted, and she was immediately transferred to the operating room for repair. During the procedure, an anomalous RCA was discovered with its origin in the dissected tissue, which was initially ligated and then bypassed using greater saphenous vein graft.

## Introduction

Anomalous aortic origin of the right coronary artery (AAORCA) is an anatomic irregularity with the right coronary artery (RCA) arising from the left sinus of Valsalva and has a prevalence of ~1% in the general population [1]. While most patients with AAORCA are asymptomatic, approximately half of patients can have devastating manifestations, including myocardial infarction, arrhythmias, and sudden cardiac death [1–4]. AAORCA is being more frequently diagnosed incidentally with advances in imaging modalities [2, 3]. Specifically, coronary computed tomography angiography (CCTA) provides proper visualization of coronary artery anatomy. Although angiocardiography is considered the gold standard diagnostic imaging modality, it is not the first-line approach for AAORCA diagnosis due to its invasive nature [4, 5].

CCTA also detects clinically significant features of AAORCA [6]. Regarding arterial diameters, CCTA allows assessment of any vessel narrowing. This imaging modality also enables the evaluation of morphology of the proximal arterial segment, which is categorized as normal, oval-shape, or slit-like (≥50% decrease in minimum diameter) [6]. In addition, CCTA can detect acute angularity of the RCA take-off, any intramural arterial course, origin of the artery in relation to the aortic commissure and type of ostia [6, 7]. These anatomic characteristics are used to stratify the risk of patients with AAORCA, ultimately determining optimal management [8]. Patients are considered to be high-risk if they are symptomatic, have AAORCA with interarterial or intramural components, or have evidence of myocardial ischemia [9]. These patients require surgical intervention and physical activity restriction [4].

Despite ample data addressing AAORCA diagnosis and management, there is a paucity of literature regarding intraoperative findings of AAORCA during emergent cardiac surgery. This report highlights the discovery and intervention of AAORCA during emergency surgery for type A aortic dissection.

## Case report

**Figure 1:**
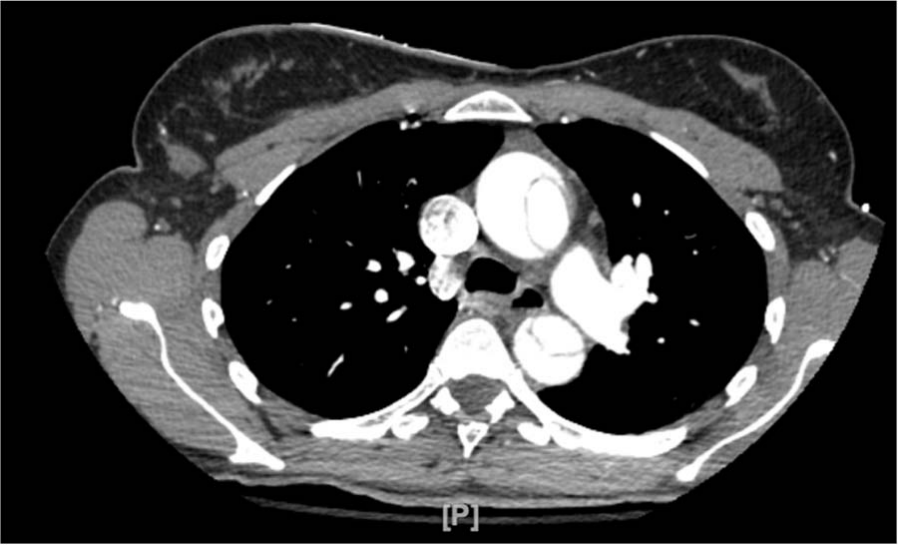
CTA demonstrating type A aortic dissection, with false lumen present in both the ascending and descending portions of the aorta.

A 48-year-old female patient with no past medical history presented for the evaluation of sudden-onset chest pain. An electrocardiogram was performed, revealing ST segment elevation in the aVR lead and diffuse ST segment depressions in the anterolateral leads. During coronary angiography, the left and right main coronary arteries were unable to be engaged. An aortogram was then conducted, which raised concern for an aortic dissection as a false lumen was identified. The procedure was aborted and a computed tomography angiogram (CTA) was obtained that showed a type A aortic dissection along with aneurysmal dilatation of the ascending thoracic aorta (Fig. 1). Cardiothoracic surgery then took the patient emergently to the operating room.

After cardiopulmonary bypass was achieved and the patient was cooled to 18°C, a cross-clamp was placed at the mid-ascending aorta, and the aorta was entered. Cardioplegia solution was delivered through the left main coronary ostium. However, the right main coronary artery ostium was unable to be visualized. After further inspection, the right main coronary artery was noted to have an intramural course through the wall of the aorta in the direction of the left coronary sinus. The dissected edges of the right main coronary artery were debrided, and cardioplegia solution was delivered through it.

Assessment of the aortic valve revealed no significant disease and the aortic dissection extended to the annulus along the length of the non-coronary and right sinuses. The dissected portion of the aorta was excised from the level of the sinutubular junction to the area just proximal to the cross-clamp. After starting retrograde cerebral perfusion, the cross-clamp was removed and the remaining part of the ascending aorta was resected, revealing a circumferential dissection. While back-bleeding was noted from the three arch vessels, there was a tear around the base of the innominate artery, precluding a hemi-arch repair. At this time, the innominate artery was circumferentially excised off the aortic arch, and tissue was resected from its base until intact intima was visualized. A Hemashield graft was then anastomosed to the remaining part of the aortic arch, and the single-side arm of the graft was anastomosed to the innominate artery.

When reassessing the aortic valve and root, it was determined that there was normal and intact tissue. The decision was made to preserve the aortic valve via a resuspension technique, and the proximal end of the graft was anastomosed to the sinotubular junction. An attempt was made to anastomose the RCA to the side of the aortic graft. However, due to its involvement with the aortic dissection and inadequate length, this was unsuccessful. As such, the proximal native RCA was ligated, and a single coronary artery bypass was performed utilizing the greater saphenous vein. Afterward, the patient was weaned from cardiopulmonary bypass. The chest cavity was closed and the patient was transferred to the intensive care unit.

The following day, an echocardiogram was performed, which revealed decreased left ventricular ejection fraction and mild aortic regurgitation. On postoperative Day 4, the patient was discharged home while continuing cardiac rehabilitation.

## Discussion

In 1–2% of cases, patients with a type A aortic dissection also have an acute myocardial infarction. This may be due to the proximal aspect of the dissection compressing a coronary artery [10–12]. However, the association between acute aortic dissection and myocardial infarction may be increased in patients with AAORCA secondary to characteristics such as slit-like proximal arterial segment, vessel narrowing near the origin, intramural course, and an acute take-off angle [13, 14]. Furthermore, AAORCA in the setting of aortic dissection may increase the technical difficulty of replacing the aortic root [11–14].

This report illustrated that cases of acute aortic dissection associated with AAORCA represent life-threatening circumstances, as there is a higher propensity of these patients also having a myocardial infarction. Although the RCA was not fully examined as it was incorporated into the dissected tissue, it had an intramural course through the wall of the aorta, which is a notable characteristic of AAORCA increasing the risk of myocardial infarction [11–13]. Although literature describing the lethal complications associated with AAORCA exists, this report demonstrates a rare presentation of this phenomenon.

Currently, genetic inheritance of coronary arterial anomalies is being investigated, which may provide insight into screening guidelines of relatives with AAORCA [15]. These measures can hopefully enable providers to identify patients with AAORCA and ultimately prevent fatal complications.

## Conflict of interest statement

None declared.

## Funding

None declared.
